# Effects of Interspecific Chromosome Substitution in Upland Cotton on Cottonseed Micronutrients

**DOI:** 10.3390/plants9091081

**Published:** 2020-08-23

**Authors:** Nacer Bellaloui, Sukumar Saha, Jennifer L. Tonos, Jodi A. Scheffler, Johnie N. Jenkins, Jack C. McCarty, David M. Stelly

**Affiliations:** 1 Crop Genetics Research Unit, USDA, Agriculture Research Service, 141 Experiment Station Road, Stoneville, MS 38776, USA; jennifer.tonos@usda.gov (J.L.T.); Jodi.Scheffler@ars.usda.gov (J.A.S.); 2 Genetics and Sustainable Agriculture Research Unit, USDA, Agriculture Research Service, 810 Highway 12 East, Starkville, MS 39762, USA; sukumar.saha@ars.usda.gov (S.S.); johnie.jenkins@usda.gov (J.N.J.); jack.mccarty@usda.gov (J.C.M.); 3 Department of Soil and Crop Sciences, Texas A&M University, College Station, TX 77843, USA; stelly@tamu.edu

**Keywords:** cottonseed nutrition, micronutrients, chromosome substitution, cotton, mineral nutrition

## Abstract

Micronutrients are essential for plant growth and development, and important for human health nutrition and livestock feed. Therefore, the discovery of novel germplasm with significant variability or higher micronutrients content in crop seeds is critical. Currently, there is no information available on the effects of chromosome or chromosome arm substitution in cotton on cottonseed micronutrients. Thus, the objective of this study was to evaluate the effects of chromosome or chromosome arm substitution on the variability and levels of micronutrients B, Fe, Cu, Zn, Mn, and Ni in cottonseed from chromosome substitution (CS) cotton lines. Our hypothesis was that interspecific chromosome substitution in cotton can affect cottonseed micronutrients content, resulting in significant differences and variabilities of these nutrients among CS lines and between CS lines and the controls. Nine CS lines were grown in two-field experiments at two locations (in 2013 in South Carolina, USA; and in 2014 in Mississippi, USA). TM-1 (the recurrent parent of the CS line) and AM UA48 (cultivar) were used as control. The results showed significant variability among CS lines compared to the controls AM UA48 and TM-1. For example, in South Carolina (SC), B concentration in cottonseed ranged from 10.35 mg kg^−1^ in CS-M02 to 13.67 mg kg^−1^ in CS-T04. The concentration of Cu ranged from 4.81 mg kg^−1^ in CS-B08sh to 7.65 mg kg^−1^ in CS-T02, and CS-T02 was higher than both controls. The concentration of Fe ranged from 36.09 mg kg^−1^ to 56.69 mg kg^−1^ (an increase up to 57%), and six CS lines (CS-B02, CS-B08sh, CS-M02, CS-M04, CS-T02, and CS-T04) had higher concentration than both controls in 2013. In 2014 at the Mississippi location (MS), similar observation was found with CS lines for micronutrients content. The CS lines with higher concentrations of these micronutrients can be used as a genetic tool toward QTL identification for desired seed traits because these lines are genetically similar with TM-1, except the substituted chromosome or chromosome segment pairs from the alien species. Chromosome substitution provides an effective means for upland cotton improvement by targeted interspecific introgression, yielding CS lines that facilitate trait discovery, such as seed micronutritional qualities, due to increased isogenicity and markedly reduced complexity from epistatic interactions with non-target alien chromosomes. The positive correlation between B, Cu, and Fe at both locations, between Ni and Mn, between Zn and Cu, and between Zn and Ni at both locations signify the importance of a good agricultural and fertilizer management of these nutrients to maintain higher cottonseed nutrient content.

## 1. Introduction

Micronutrients are essential for plant growth, plant development, human health nutrition, and livestock feed [1]. Malnutrition due to micronutrient deficiencies, including Zn and Fe, still exists in several developing countries [2,3,4] in spite of crop fortification [5,6]. Food staple crops are often deficient in micronutrients such as Fe and Zn [7], causing mineral malnutrition, affecting more than two billion people globally. The Food and Agriculture Organization of United Nations reported [8] that The State of Food Security and Nutrition in the World committed to ending hunger and malnutrition worldwide by 2030, as adequate levels of micronutrients, such as zinc nutrition, are critical for proper growth [9], immunocompetence and neurobehavioral development [3], normal pregnancy outcome and child growth, immune function, and neurobehavioral development [10,11]. As reported by others [12], one of the challenges to access required nutrients by low-income population is to develop a sustainable and cost-effective way to deliver the required nutrients. Seed, of course, are an excellent delivery vehicle as feed and food. Therefore, identifying crop germplasm with high seed nutritional contents of micronutrients, including B, Zn, Fe, Cu, Mn, and Ni, is critical and considered as a major goal in crop breeding programs and crop fortification. Reports have shown that micronutrient deficiencies of Fe and Zn still result in serious health issues in pregnant women and children below five years of age [13]. Iron deficiency leads to anemia of 24.8% of the global population and more than 65% of the preschool-aged children in Africa and southeast Asia [14]. Zinc deficiency results in public health risk and is responsible for the annual mortality of 433,000 children under the age of five [15].

One of the strategies to enhance the content of micronutrients, including Fe and Zn, is to increase agricultural crops through breeding improvement [9] and genetic biofortification, which is one of the most cost-effective and sustainable approaches [4,9]. Molecular genetics and genomic approaches have been previously used to identify genes controlling minerals accumulation such as selenium [16] and Zn [17]. Other researchers reported genes that were differentially expressed when comparisons of hyperaccumulators and nonhyperaccumulators were used [18]. Quantitative trait locus (QTL) effects on minerals were by analysis in beans [19,20], rice [21], in *Arabidopsis* [22], and soybeans [23]. Although some candidate genes were identified for many of the QTLs based on gene function, other QTLs have clear candidates, indicating that new genes controlling mineral uptake or translocation are yet to be identified [7,24].

The accumulation of minerals in seed involves genetic and several processes, including nutrient uptake, translocation, redistribution, and accumulation [6,25], and most of the genetic bases of these process are not well understood [26]. The physiological and metabolic roles of micronutrients such as B, Fe, Cu, Mn, Zn, and Ni in plants are well documented [27,28], and molecular functions of micronutrients have also been studied. For example, overexpression of the B transporter for the B efflux, BOR1, for xylem loading [29], and a major intrinsic protein, NIP5;1, for B uptake [30], were studied. Further, cloning BOR1-like homologs in *Brassica napus* BnBOR1;3a and BnBOR1;3c, the expression of BnBOR1;1c and BnBOR1;2a induced by B deficiency [31], and the transcription factor gene WRKY6 in *Arabidopsis thaliana* [32] were also reported. For example, QTL analysis for mineral accumulation in seeds may facilitate identifying genes encoding transporters, chelators, biosynthesis enzymes, and regulatory factors, including protein kinases, membrane receptors, and transcription factors [33], were also reported. Quantitative trait loci associated with mineral accumulation in crop seeds were identified in rice [34] and wheat [35]. However, limited QTLs were identified for Fe, Zn, and Mn, B, and Cu Sankaran [23]. QTLs for Mn, Fe, Zn, B, and Cu were identified in *Brassicaceae* shoots [36,37] and QTLs for Fe efficiency were identified in soybean [38].

Although QTL mapping is a powerful tool to study complex traits such as seed mineral contents [39,40] to identify genomic regions related for a trait variation based on the association between polymorphic markers and phenotypic measurements, its use is still limited. When interspecific introgression is involved, it may be especially valuable to create isogenic chromosome substitution (CS) and/or chromosome segment substitution (CSS) lines. In these lines, a pair of chromosomes or chromosome segments have been substituted with a pair of chromosomes or chromosome segments from another related species. All the lines have the same parental background, with only the substituted pair of chromosomes or chromosomal segment being different. Because of the way they were developed, CSS lines can concomitantly accomplish germplasm introgression, experimentally facilitate trait detection, crudely localize genetic effects, and serve as isogenic parents for further genetic dissection, mapping and breeding [41,42]. The crop improvement of nutrient uptake and use efficiency to avoid nutrients deficiencies in seeds has been among the major goals in crops mineral nutrition breeding. Efficient selection for a nutrient trait depends on heritability, total variance due to genetic variance, and variance due to environment. Selection efficiency can be improved by associating key chromosomal regions with molecular markers and rendering them amenable to marker-assisted selection. Therefore, the development of other genetic techniques, such as chromosomal substitution, genotyping, and association genetics related to genetic regions related to the target complex trait, may further facilitate breeding selection efficiencies [43].

The development of cotton lines that seed-accumulate micronutrients would constitute new genetic resources for potential improvements of cottonseed feed that enhance domesticated animal health nutrition. Changes that improve uptake from micronutrient-deficient soils could improve crop growth and production. The genetics of cottonseed traits of chromosome substitution germplasm lines (CS) is scarce [44], and information on the effects of chromosome substitution or chromosome arms on micronutrients in cottonseed is not available [45]. Therefore, the objective of this research was to evaluate cotton chromosome substitution germplasm lines for micronutrients. The CS lines are near-isogenic to each other and the recurrent parent TM-1 with one chromosome or chromosome arm introgressed [46,47,48,49,50,51]. We used chromosome comparison (specific chromosomes or chromosome arms in the TM-1 background) to evaluate higher CS lines as the comparative method can result in seed nutritional quality traits improvement [45].

## 2. Results

### 2.1. Analysis of Variance and Mean Values

Analysis of variance showed that location, line (CS line, TM-1, or check UA 48), and their interactions were significant for B, Cu, Fe, Mn, Ni, and Zn (Table 1). The significant effects of the interaction between location and line indicated that the ranking of these minerals changed depending on the environment of that location (Table 1), especially drought, temperature, and soil fertility.

In South Carolina, the mean values showed that B concentration was higher in three CS lines (CS-B04, CS-M08sh, and CS-T04) (Table 2). Boron concentration ranged from the lowest (10.35 mg kg^−1^) in CS-M02 to the highest (13.67 mg kg^−1^) in CS-T04 (an increase from 0% to 32%). Copper (Cu) concentration ranged from the lowest 4.81 mg kg^−1^ in CS-B08sh to highest (7.65 mg kg^−1^) in CS-T02 (an increase ranged from 0% of 59%), and CS-T02 was the only CS line that showed superiority which was higher than both controls AM UA48 and the parent TM-1. Iron concentrations ranged from 36.09 mg kg^−1^ to 56.69 mg kg^−1^ (an increase from 0 %to 57%), and six CS lines (CS-B02, CS-B08sh, CS-M02, CS-M04, CS-T02, and CS-T04) had higher concentration than both controls. Manganese concentrations ranged from the lowest (13.72 mg kg^−1^) in CS-M02 to 21.13 mg kg^−1^ in CS-T04 (an increase from 0% to 54%), while CS-T02 and CS-T04 showed higher Mn concentrations than both controls. Concentrations of Ni ranged from 1.63 mg kg^−1^ in CS-M04 to 2.86 mg kg^−1^ in CS-T04 (an increase from 0% to 54%), and CS-T04 was found to be higher than both controls. Eight CS lines were found to be higher in Zn concentrations than the cultivar AM UA48 and TM-1 (Table 2).

In Mississippi, mean values showed that the B ranged from 11.24 mg kg^−1^ (CS-T02) to 15.16 mg kg^−1^ (CS-M08sh), which was the only CS line that showed superiority of B concentration than both controls (Table 3). The concentration of Cu ranged from the lowest (4.43 mg kg^−1^) in CS-T02 to the highest (6.68 mg kg^−1^) in CS-M04. CS-M04 showed superiority in Cu concentration than all CS lines and controls. CS-M02, CS-M04, CS-M08sh, CS-T04, CS-T08sh showed higher Cu concentration than the cultivar control AMUA48 and competitive to TM-1. The concentration of Fe was higher in all nine CS lines than the controls. Concentration of Fe ranged from the lowest (41.18 mg kg^−1^ in TM-1 and 41.28 mg kg^−1^ in AM UA48) to the highest (49.75 mg kg^−1^) in CS-T04 (an increase from 0 to 20.8%). Mn showed significant variability among CS lines and compared with the controls AM UA48 and TM-1. The concentration of Mn ranged from the lowest (13.05 mg kg^−1^) in CS-T02 to the highest (20.01 mg kg^−1^) in CS-T04 (an increase from 0% to 53%). Four CS lines (CS-M02, CS-M08sh, CS-T04, and CS-T08sh) showed higher Mn concentration than AM UA48 and TM-1. Eight CS lines (CS-B02, CS-B04, CS-B08sh, CS-M02, CS-M04, CS-M08sh, CS-T04, and CS-T08sh) showed high levels of Ni concentrations than TM-1, but only one CS line (CS-T08sh) showed higher Ni concentration than both controls. Concentration of Zn ranged from 48.76 mg kg^−1^ in CS-B04 to 98.28 mg kg^−1^ in CS-T04 (an increase from 0% to 101.6%), and three CS lines (CS-M04, CS-M08sh, CS-T04) showed superiority in Zn content than AM UA48 and TM-1 (Table 3).

### 2.2. Correlation and Distribution of Micronutrients

**Figure 1:**
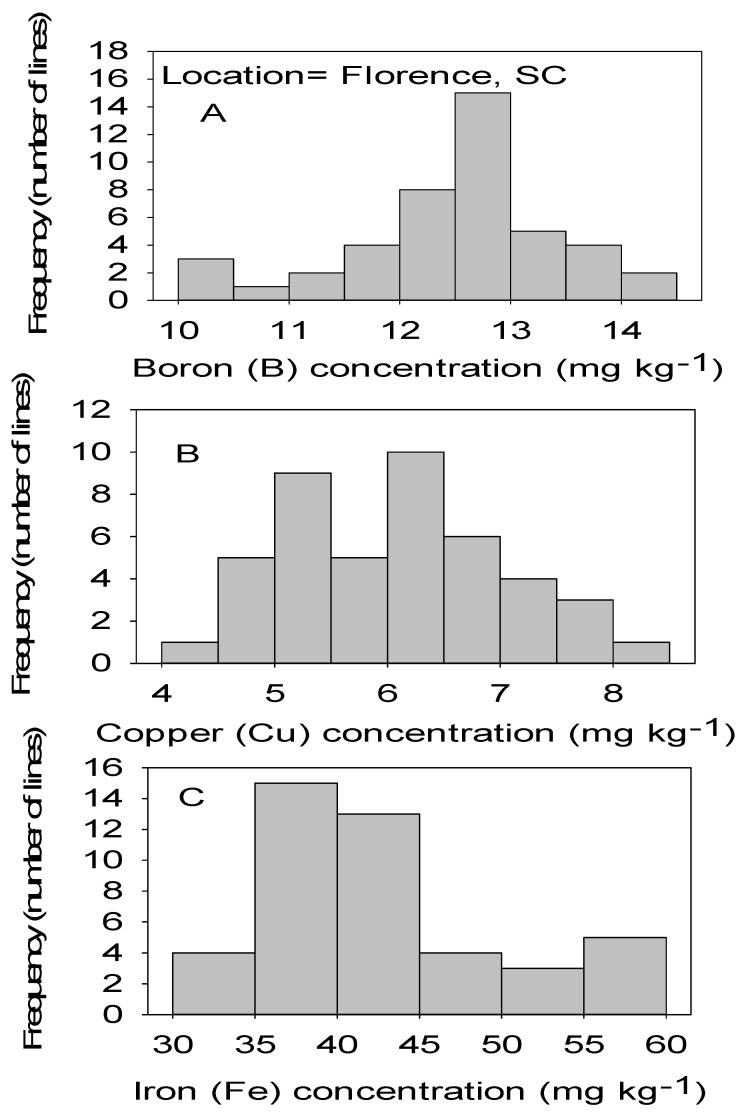
Distribution of micronutrients B (**A**), Cu (**B**), and Fe (**C**) in cottonseed in CS lines in cotton in Florence, SC, USA.

**Figure 2:**
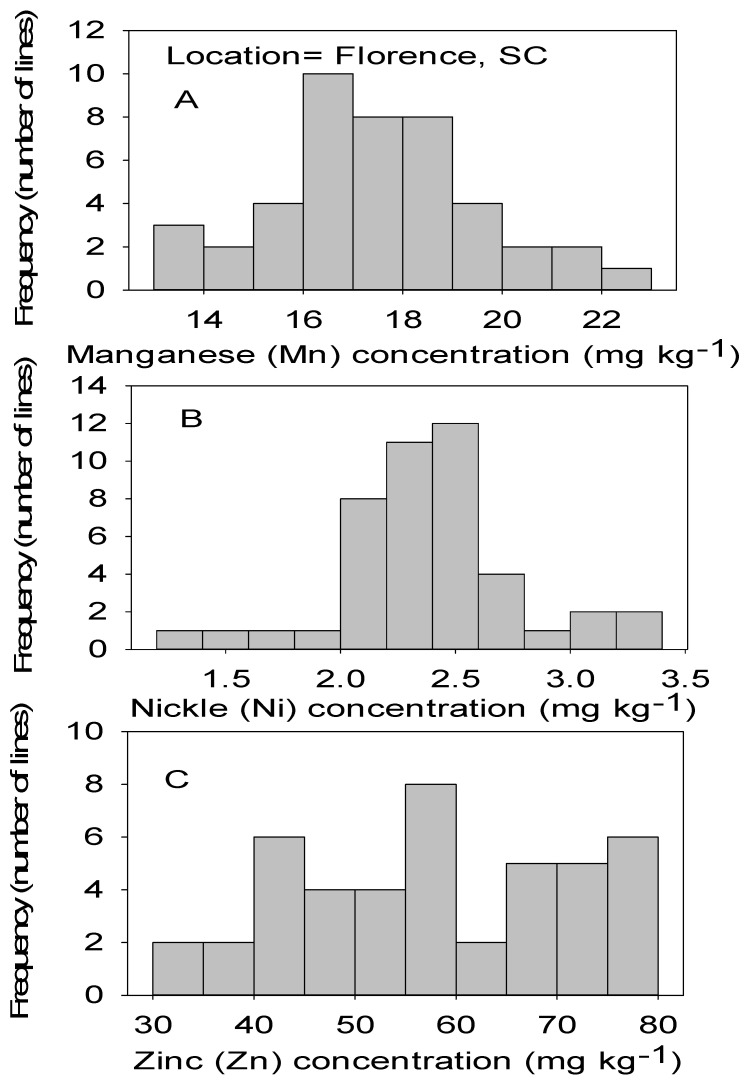
Distribution of micronutrients Mn (**A**), Ni (**B**), and Zn (**C**) in cottonseed in chromosome substitution lines (CS) in cotton in Florence, SC, USA.

**Figure 3:**
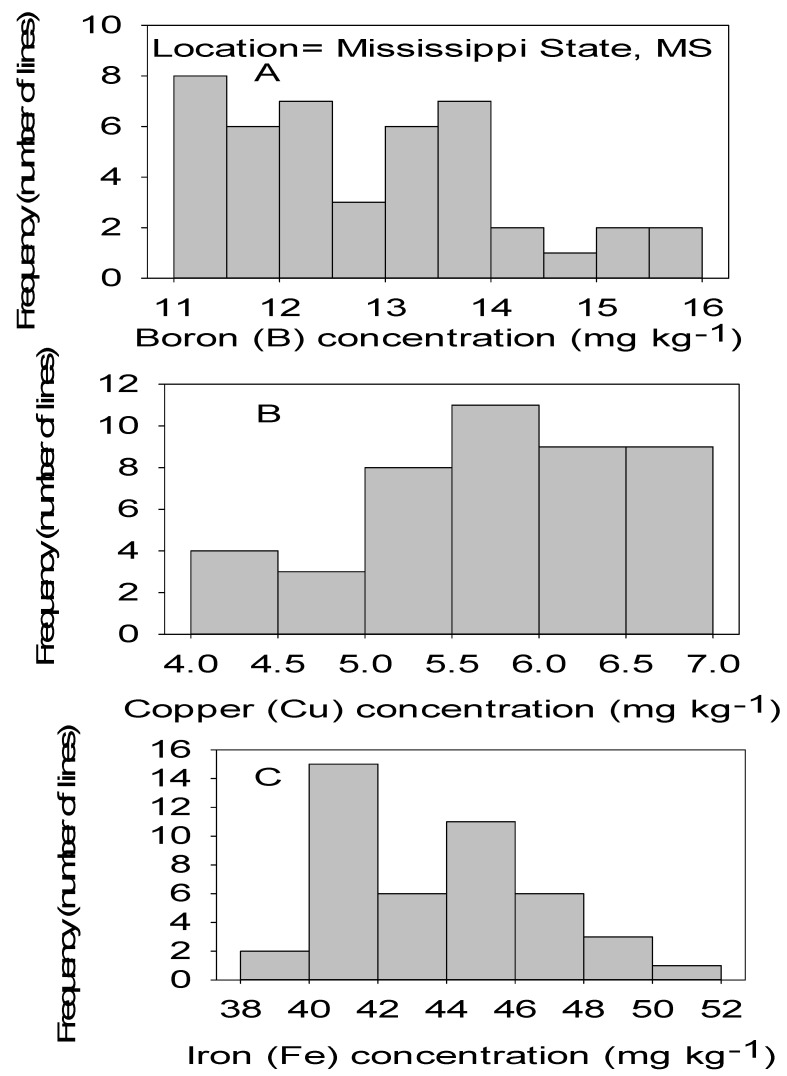
Distribution of micronutrients B (**A**), Cu (**B**), and Fe (**C**) in cottonseed in CS lines in cotton in Mississippi, MS, USA.

**Figure 4:**
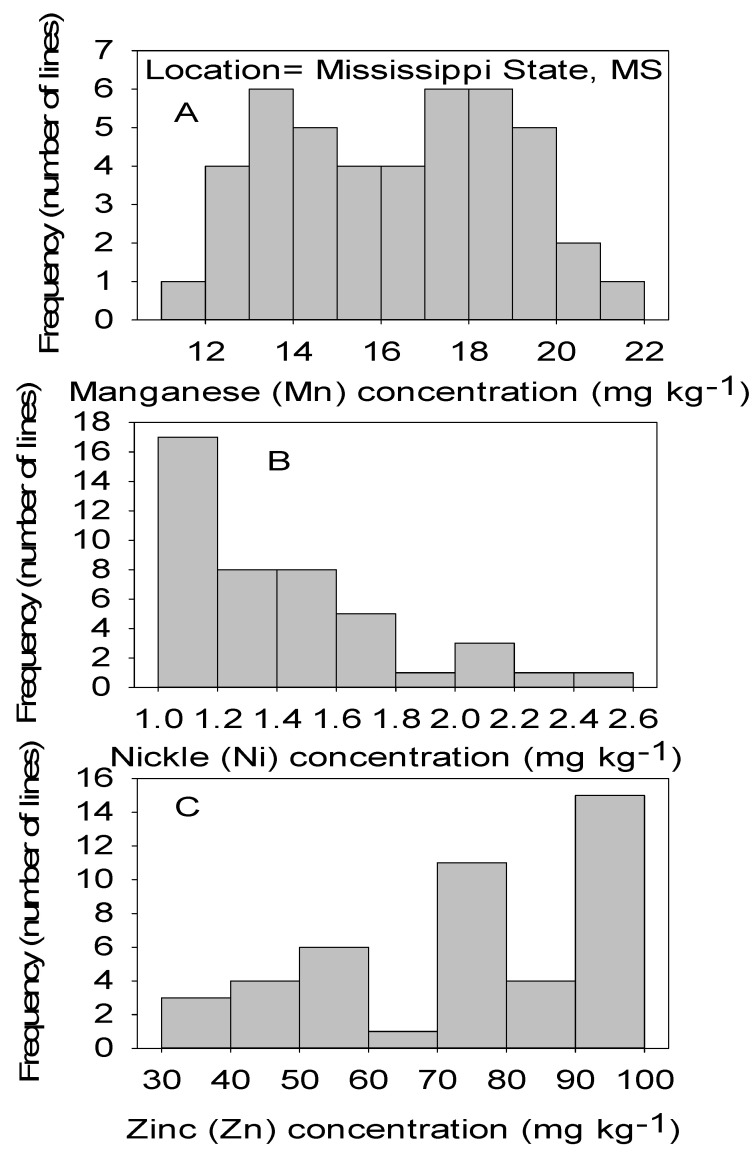
Distribution of micronutrients Mn (**A**), Ni (**B**), and Zn (**C**) in cottonseed in CS lines in cotton in Mississippi, MS, USA.

Correlation analysis showed that Mn had significant positive correlations with B, Cu, and Fe in both locations (Table 4 and Table 5). Also, significant positive correlation between Ni and Mn, between Zn and Cu, and between Zn and Ni in both locations. No correlation was found between Cu and B, between Fe and B, between Ni and Fe, and between Mn and Zn in both locations. Positive correlation was found between Zn and B in MS only, between Ni and B in NC only, and between Ni and Cu in SC only (Table 4 and Table 5). Figure 1, Figure 2, Figure 3 and Figure 4 show significant distribution and a wide variability of minerals reflected by a normal probability distribution and bimodal, which indicate that the distribution of minerals in CS lines is complex and needs further research.

## 3. Discussion

Cotton chromosome substitution germplasm lines, near-isogenic to the recurrent parent TM-1, an inbred line, with one chromosome or chromosome arm introgressed from *G. barbadense* L. line 3-79, have been used previously to associate seed traits with chromosomes or chromosome arms [46,47,48,49,50,51]. However, the genetics of cottonseed traits, including seed nutrition of chromosome substitution germplasm lines, are scarce [44]. Previous research was conducted to identify desirable cottonseed traits in five commercial cultivars, 13 CS lines, 3-79 (donor parent), TM-1 (recurrent parent), and F3 hybrids [49,50] using chromosome associations with seed traits by the comparative method. The authors concluded that using chromosome substitution germplasm lines should provide valuable genetic information for desirable seed trait analysis in other crops and provide useful genetic information to breeders to select for cottonseed nutritional qualities. Currently, there are no reports of chromosome substitution effects on micronutrients in cottonseed. However, there are examples from research on other seed nutrients such as protein and oil. For example, the authors of [45] conducted an experiment to study the effects of chromosome substitution on seed for F3 hybrids of 13 cotton chromosome substitution lines crossed with five elite cultivars grown in four environments. They found significant additive effects for protein and oil content, demonstrating that seed traits conferring nutritional qualities can be genetically improved using chromosome substitutions. Also, it indicated that chromosome associations (specific chromosomes or chromosome arms in the TM-1 background), using a comparative method, can result in seed nutritional quality trait improvement [45,51].

Our research showed that several chromosome substitution lines had exhibited higher levels of some micronutrients in cottonseed compared to the parent TM-1. For example, almost all of the CS lines, except CS-T08sh, had higher concentrations of Fe and Zn in cottonseed grown in South Carolina compared to the parent TM-1, suggesting the potential association of the alien species substituted chromosome, causing an increase in Fe and Zn concentration at this location (Table 2). Our results showed that Cu concentration in CS-T02 line was significantly higher in both locations. Similar observations were observed for Fe concentration in CS-T04 line and Mn in CS-T04, showing the superiority of these CS lines for these microminerals under both environments. In South Carolina, Fe showed superiority in six lines (CS-B02, CS-B08sh, CS-M02, CS-M04, CS-T02, and CS-T04), and the concentrations in these CS lines were also higher than both controls. In Mississippi, four CS lines (CS-M02, CS-M08sh, CS-T04, and CS-T08sh) showed higher Mn concentration than controls and eight CS lines (CS-B02, CS-B04, CS-B08sh, CS-M02, CS-M04, CS-M08sh, CS-T04, and CS-T08sh) showed higher levels of Ni concentrations than TM-1, while only CS-T08sh showed higher Ni concentration than controls; three CS lines (CS-M04, CS-M08sh, CS-T04) showed superiority in Zn content compared to AM UA48 and TM-1. Although there is no information on the effects of chromosome substitution on cottonseed micronutrients, there are reports about other nutrients such as protein and oil and other traits in CS lines or other populations. For example, homozygous and heterozygous dominance effects and additive effects on cottonseed protein and oil were found [45]. They authors reported that 9 out of 20 parents showed significant negative homozygous dominance effects for oil content, but four parents were positive. It was found that chromosomes 2, 7, 18, 25, and chromosome arm 5sh of 3-79 donor parent were associated with increased seed oil content in DP90, showing positive heterozygous dominance effects. However, chromosome arms 22sh and 22Lo were associated with reduced seed oil content, showing negative heterozygous dominance effects [45]. Similar effects were shown for SG747, where heterozygous dominance effects for chromosomes 2 and 17 and chromosome arms 15sh, 22sh, and 22Lo were associated with higher seed oil content, whereas chromosomes 16 and 25 were associated with reduced oil content [45]. Researchers working on other genetic materials showed that mineral nutrient concentrations were significantly improved in a RIL population compared to the parents. They also concluded that rich genetic diversity for mineral nutrient concentrations can exist and provide novel alleles for higher grain mineral concentrations [52]. Our results also showed that some CS lines exhibited increased B, Fe, Mn, Zn, and Ni in one location (Mississippi), but not in the other location (South Carolina), indicating that these micronutrients responded positively under the environmental conditions of the Mississippi location in some CS lines, but not others.

The current research demonstrated that chromosome substitution resulted in higher concentration of micronutrients in some CS lines, but in others no change or reduced concentrations were observed. The positive or negative response of these micronutrients was dependent on location and CS line. The CS lines with higher concentrations of these micronutrients can be used as parents for breeding programs to increase cottonseed nutritional qualities. The results showed that identification of chromosome associations with high seed nutritional quality traits is possible and these high micronutrients CS lines can be crossed to elite germplasm and cultivars [52]. Although the genetic system for cottonseed traits is complicated and multiple genetic models are involved in cottonseed traits [53,54,55], these complicated genetic models can be useful to identify desirable cottonseed traits [45,56,57].

CS lines with higher micronutrients concentrations in seed reflect nutrient efficiency for these nutrients and may allow the plant to overcome deficiencies of these nutrients in soil. It was reported that when genotypes grown on soils with low micronutrient availability due to chemical or biological fixation, or spatial or temporal unavailability, micronutrient-efficient genotypes have greater yield in comparison to inefficient genotypes even when fertilized with smaller amounts or less frequently [58]. The importance of genotype on differential micronutrient efficiency has been previously reported and discussed in detail [59,60,61]. The current research demonstrated that several CS lines had higher concentration of micronutrients compared to the parent TM-1 line, suggesting the potential association of the substituted chromosome or chromosome segment pair of the traits of interest. The high demand for cottonseed derived products for nutritional and industrial applications highlights the urgent need to unveil the genetic mechanism associated with the trait of interest. Micronutrients in cottonseed are very important considering cottonseed is one of the most important natural sources of feed livestock. To our knowledge, this is the first report on the potential effects of chromosomal substitution on specific micronutrients in cottonseed. The CS lines with improved concentrations of these micronutrients can be used as parents for further breeding programs to improve the cottonseed nutritional qualities.

The positive correlation between B, Cu, and Fe in both locations, between Ni and Mn, between Zn and Cu, and between Zn and Ni in both locations signify the positive interactions between these nutrients, indicating the possibility of the increase of these nutrients through uptake and transport that leads to the increase of the other. The positive correlation of some nutrients, such as between Zn and B in MS only, between Ni and B in NC only, and between Ni and Cu in SC only, indicate that the relationships between these nutrients are dependent on the environmental conditions of that location and the CS line. It was reported that the concentrations of elements in grain are also influenced by complex genetics and environmental factors [4].

Although the genetic system for cottonseed traits is complicated and multiple genetic models have been used to analyze cottonseed traits [54,55,56], some of these genetic models may be useful to dissect the complex cottonseed quality traits [45,56,57]. For example, the CS lines were used to unveil the genetic mechanism associated with the nutritional quality of oil and protein percentage in cotton seed, and this approach can be applied to seed micronutrients [52]. Although the genetic controls of these micronutrients in cottonseed are unknown and inferably complex, loci exerting major effects should be detectable using methods similar to others [45,46,47,48,49,50,51]. By applying the genetic AD model system using the CS lines, the effect of an individual micronutrient can be genetically dissected into additive/dominance effects. More refined localization should be possible using chromosome specific recombinant inbred lines (CSRIL) populations from the intercrosses of TM-1 (*G. hirsutum*) with the CS lines of interest, followed by inbreeding, e.g., by the single seed decent method [62,63]. These CSRIL populations, each specific to a CS line, provides a powerful analytical tool to dissect many complexly inherited traits. For cottonseed micronutrients, further genetic analysis and molecular mapping are needed. The CSRIL approach also provides a means for targeted introgression of a trait of interest and greatly reduces the linkage drag effects by undesirable genes from the alien species into Upland cotton. Using molecular markers linked to desirable donor genes will provide breeders means to introduce novel improvements into Upland cotton as part of their improvement programs.

In summary, this research provided valuable information towards the effort to improve micronutrient content in Upland cottonseed. This study is important from the following perspectives: (1) It reported, for the first time, information on the chromosome or chromosome segments from the wild species *G. tomentosum* and *G. mustelinum* with improved micronutrient content; (2) it discovered specific chromosomal association with the cottonseed micronutrient content gene(s); and (3) it developed some novel germplasm with the gene(s) for improved micronutrient content from interspecific crosses using the wild species.

## 4. Materials and Methods

### 4.1. Chromosome Substitution Cotton Lines (CS)

Nine euploid (2*n* = 52) cotton chromosome substitution lines were used. They were represented by the substitution of chromosome 2, chromosome 4, and the short arm of chromosome 8 (8sh) from three tetraploid species of *G. barbadense* (CS-B), *G. tomentosum* (CS-T), and *G. mustelinum* (CS-M), respectively, into an Upland cotton (TM-1, *G. hirsutum*) genetic background. TM-1, the recurrent parent of the CS lines, and ‘AM UA48’, Reg. No. CV-129, [64] a cultivar with yield and quality traits typical for upland cotton, were used in this research as controls. The nine CS lines were CS-B02, CS-B04, CS-B08sh, CS-M02, CS-M04, CS-M08sh, CS-T02, CS-T04, CS-T08sh. These lines were grown in two locations: Florence, South Carolina (SC, 34.1° N 79.4° W) in 2013, and Mississippi State, Mississippi (MS, 33.4° N 88.8° W) in 2014. The SC soil type was a Norfolk loamy sand (fine-loamy, kaolinitic, thermic typic Kandiudults). The MS soil type was a Leeper silty clay loam (fine, smectitic, nonacid, thermic Vertic Epiaquept). Each entry was grown in 12-m-long single-row plots with rows spaced 97-cm apart and plants spaced 10-cm apart (about 110 plants per row). A 25-open-pollinated-boll sample per plot was hand-harvested at both locations from the first fruit position near the middle node of a plant to determine fiber properties. Samples were ginned on a 10-saw laboratory gin to separate seeds from the lint. Seeds from these samples were used for micronutrient analysis.

### 4.2. Seed Mineral Nutrient Analyses

Mineral concentrations in Zn, Cu, and Ni were determined in the ground, dried seed samples. Briefly, samples were ground with a Laboratory Mill 3600 (Perten, Springfield, IL, USA), and analyzed by digesting 0.5 g of dried ground seed in HNO_3_ in a microwave digestion system. Concentrations were determined using inductively coupled plasma spectrometry (Thermo Jarrell-Ash Model 61E ICP and Thermo Jarrell-Ash Autosampler 300) (Thermo Jarrell-Ash Corporation, Waltham, MA, USA) [65]. Seed B and Fe were determined as detailed below.

### 4.3. Determination of Seed B and Fe

Boron concentration in mature seeds was determined by the azomethine-H method [66,67]. Briefly, seed samples were ground by a Laboratory Mill 3600 (Perten, Springfield, IL, USA) and a 1.0-g sample was combusted to ash at 500 °C and extracted with 20 mL of 2-M HCl at 90 °C for 10 min. Then, the mixture was filtered, and a 2 mL sample added to 4 mL of buffer solution containing 25% ammonium acetate, 1.5% EDTA, and 12.5% acetic acid. A freshly prepared solution (4 mL) of 0.45% azomethine-H in 1% of ascorbic acid [68] was added, and the B concentration was determined at 420 nm using a Beckman Coulter DU 800 spectrophotometer (Beckman Coulter, Inc., Brea, CA, USA). Determination of Fe concentration in mature seeds was conducted according to established methods [69,70]. Briefly, seed samples were ground using a Laboratory Mill 3600 (Perten, Springfield, IL, USA) as described above. Then, samples were digested with hydrochloric acid (109 mL of 3% w/w) and extracted. Determination of Fe concentration was conducted by the color complex reaction between ferrous Fe with 1,10-phenanthroline as reported by others [65,69,70,71]. Phenanthroline reagent solution of 0.25% (w/v) in 25% (v/v) ethanol and quinol solution (1% w/v) were prepared, and the concentrations of Fe ranging from 0.0 μg∙mL^−1^ to 4 μg∙mL^−1^ of Fe in 0.4-M HCl were made for the standard curve. Iron concentration was measured by a Beckman Coulter DU 800 spectrophotometer at an absorbance of 510 nm as previously described by others [65,69,70,71].

### 4.4. Experimental Design and Statistical Analysis

Cotton genotypes were grown in a randomized complete block design with four replications within each location. PROC MIXED (SAS, SAS Institute, 2002–2010) was conducted to evaluate the effects of location, line, and their interactions. Replicates within location were considered as random effects. Line and location were considered as fixed effects. Multiple-comparison procedures (mean separation test) were conducted at significance level of 5% in SAS (SAS, SAS Institute, 2002–2010) [72]. Since location-by-line interactions were significant for micronutrients, results were presented separately by location. Significant differences in nutrients between a specific CS line and TM-1 were considered as due to the specific substituted chromosome or chromosome arm from the donor parent of *G. barbadense*, *G. tomentosum,* and *G. mustelinum* because each the genetic background of each CS line is expected to be nearly isogenic (~98.6%) to TM-1. Correlation was analyzed in SAS using Proc Corr (SAS, SAS Institute, 2002–2010) [72].

## 5. Conclusions

Micronutrient deficiencies constrain health and development of humans and animals, and cottonseed could provide a sustainable source of such micronutrients. To evaluate the possibility of enhancing micronutrient levels in cotton from wild species germplasm introgression, we used the comparative method [45] to detect potential effects of disomic chromosome substitution on seed micronutrients (B, Fe, Cu, Zn, Mn, and Ni). CS lines that exhibited higher micronutrients in both locations might be of interest as parents for genetic dissection and breeding for seed with improved micronutrient nutrition, e.g., with higher Cu concentrations, using the CS-T02 line; for higher Fe concentration, using the CS-T04 line; or for higher Mn, using CS-T04. The wide range of micronutrient levels among CS lines will be of significant interest in breeding selection for higher cottonseed nutritional qualities. This research contributes to the identification of cotton lines with higher micronutrients levels that can be used as a source for human nutrition and livestock feed. Micronutrients play an essential role in animal health, and meat and milk productivity. Deficiencies of micronutrients in animal feed lead to loss of growth sustainability, fertility, and loss of immunity, causing clinical and nonclinical diseases [73]. Therefore, maintaining optimum levels of micronutrients in animal feed, including cottonseed meal, is critical. The current research suggests that certain chromosomes of the TM-1 genome are associated with increased micronutrient levels, other chromosomes are associated with decreased micronutrients, and others do not change the level of micronutrients compared with TM-1. To our knowledge this is the first report on the effects of chromosome substitution on cottonseed micronutrients.

## Figures and Tables

**Table 1:** Analysis of variance (F and P values) of the effects of location, genotype (G) (cotton chromosome substitution (CS) lines, parent TM-1, and commercial cultivar AM UA48), and their interactions for cottonseed minerals (mg kg^−1^) in CS in different locations (Florence, SC, 2013 and Mississippi State, MS, 2014).

		B		Cu		Fe		Mn		Ni		Zn	
Effect	DF ^a^	F	P ^b^	F	P	F	P	F	P	F	P	F	P
**Location**	1	10.7	0.02	5.9	0.05	6.75	0.01	16.9	0.006	124.9	<0.0001	147	<0.0001
**Genotype**	10	44.2	<0.0001	25.5	<0.0001	54.2	<0.0001	29.4	<0.0001	10.91	<0.0001	64.7	<0.0001
Location*G	10	10.2	<0.0001	39.44	<0.0001	26.3	<0.0001	30.0	<0.0001	8.23	<0.0001	31.6	<0.0001
**Residual**		0.19		0.0822		2.98		0.73		0.043		25.0	

^a^ DF = degree of freedom. ^b,^* Significance at *p* ≤ 0.05; ** significance at *p* ≤ 0.01; *** significance at *p* ≤ 0.001.

**Table 2:** Experiment 1: Effects^†^ CS on cottonseed mineral concentrations (mg kg^−1^). The experiment was conducted in 2013 in Florence, SC, USA.

	B	Cu	Fe	Mn	Ni	Zn
**Genotype**	mg kg^−1^					
**CS-B02**	12.46 de	5.02 g	45.98 c	16.16 g	2.24 e	75.33 a
**CS-B04**	13.12 b	5.14 g	37.45 g	15.70 h	2.23 e	63.03 b
**CS-B08sh**	12.41 e	4.81 h	41.77 e	16.67 f	2.46 d	63.90 b
**CS-M02**	10.35 g	6.13 e	42.67 e	13.72 i	2.33 de	75.20 a
**CS-M04**	11.45 f	5.72 f	44.03 d	17.16 e	1.63 f	58.09 c
**CS-M08sh**	13.63 a	5.82 f	36.23 h	18.32 cd	2.42 d	53.20 d
**CS-T02**	12.43 de	7.65 a	54.77 b	19.64 b	2.60 bc	53.45 d
**CS-T04**	13.67 a	6.63 c	56.69 a	21.13 a	2.86 a	73.90 a
**CS-T08sh**	12.59 d	6.40 d	35.88 h	17.01 ef	2.63 b	35.47 g
**AM UA48**	12.77 c	6.72 c	36.09 h	18.69 c	2.60 bc	44.20 e
**TM-1**	12.35 e	7.07 b	39.33 f	18.21 d	2.45 d	42.14 f

^†^—Means within a column followed by the same letter are not significantly different at the 5% level. Test lines are lines starting with CS; AM UA48 is a check or control; TM-1 is a recurrent parent of the CS line.

**Table 3:** Experiment 2: Effects^†^ of CS on cottonseed mineral concentrations (mg kg^−1^). The experiment was conducted in 2014, Mississippi State, MS, USA.

	B	Cu	Fe	Mn	Ni	Zn
**Genotype**	mg kg^−1^					
**CS-B02**	12.80 d	5.48 e	41.81 ef	13.44 g	1.29 e	76.82 c
**CS-B04**	12.65 de	5.57 de	41.41 f	16.75 d	1.18 f	48.76 f
**CS-B08sh**	11.38 g	5.61 d	45.96 b	13.23 g	1.18 f	79.53 c
**CS-M02**	11.45 g	5.65 d	45.91 b	19.82 a	1.33 e	76.85 c
**CS-M04**	11.98 f	6.68 a	42.46 de	15.50 e	1.58 c	95.55 a
**CS-M08sh**	15.16 a	6.25 c	42.52 d	17.98 b	1.44 d	96.36 a
**CS-T02**	11.24 g	4.43 g	43.54 c	13.05 g	1.13 fg	58.30 e
**CS-T04**	13.71 c	6.21 c	49.75 a	20.01 a	1.52 c	98.28 a
**CS-T08sh**	12.43 e	6.44 b	45.60 b	18.30 b	2.15 a	39.45 g
**AM UA48**	14.35 b	4.68 f	41.28 f	17.49 c	1.83 b	61.83 d
**TM-1**	13.6 c	6.43 b	41.18 f	14.23 f	1.10 g	90.93 b

^†^—Means within a column followed by the same letter are not significantly different at the 5% level. Test lines are lines starting with CS; AM UA48 is a check or control; TM-1 is a recurrent parent of the CS line.

**Table 4:** Pearson correlation coefficients (*P* ^a^ and R values) between cottonseed minerals in CS lines grown in SC 2013.

Nutrient	B	Cu	Fe	Mn	Ni
**Cu**	R = 0.00596				
	*P* = NS ^b^				
**Fe**	R = 0.02954	0.29195			
	*P* = NS	*			
**Mn**	R = 0.59727	0.50881	0.40371		
	*P* = ***	***	**		
**Ni**	R = 0.31024	0.31225	0.2099	0.31795	
	*P* = *	*	NS	*	
**Zn**	R = −0.17679	−0.42992	0.49447	−0.25791	0.39767
	*P* = NS	**	***	NS	*

^a,^* Significance at *p* ≤ 0.05; ** significance at *p* ≤ 0.01; *** significance at *p* ≤ 0.001; ^b^ NS = not significant at 0.05 probability.

**Table 5:** Pearson correlation coefficients (*P* and R values) between cottonseed minerals in CS lines grown in MS 2014.

Nutrient	B	Cu	Fe	Mn	Ni
**Cu**	R = 0.21738				
	*P* = NS				
**Fe**	R = −0.17041	0.19739			
	*P* = NS	NS			
**Mn**	R = 0.36405	0.31066	0.41148		
	*P* = *	*	**		
**Ni**	R = 0.23750	0.21287	0.11357	0.49948	
	*P* = NS	NS	NS	***	
**Zn**	R = 0.31039	0.43265	0.15022	0.05613	−0.27541
	*P* = *	**	NS	NS	*

* Significance at *p* ≤ 0.05; ** significance at *p* ≤ 0.01; *** significance at *p* ≤ 0.001; NS = not significant at 0.05 probability.
